# Determining the efficacy of barbotage for pain relief in calcific tendinitis

**DOI:** 10.1016/j.jseint.2024.06.005

**Published:** 2024-06-19

**Authors:** W. Doug Werry, Margaret Hedeman, Arnav Sharma, John Garfi, Dmitry Elentuck, Brian Samuelsen, George Kasparyan, Mark Lemos

**Affiliations:** a Tufts University School of Medicine, Boston, MA, USA; b Yale University, New Haven, CT, USA; c Duke University School of Medicine, Durham, NC, USA; d Orthopedic Surgery Department at Lahey Hospital and Medical Center, Burlington, MA, USA; e Radiology Department at Lahey Hospital and Medical Center, Burlington, MA, USA

**Keywords:** Barbotage, Calcific tendinitis, Pain management, Aspiration and lavage, Corticosteroids, Patient-reported outcomes

## Abstract

**Background:**

Rotator cuff calcific tendinitis is a common cause of shoulder discomfort. Ultrasound-guided barbotage consists of needle aspiration and a subsequent lavage of calcium deposits in the shoulder. While barbotage has proven benefit, other options have also shown similar symptom improvement. This study aims to examine pain outcomes of patients following barbotage of calcific tendinitis. We hypothesize that barbotage will improve shoulder pain scores compared to preprocedure scores.

**Methods:**

This is a retrospective chart review of 179 ultrasound-guided barbotage interventions for calcific tendinitis of the rotator at a New England urban medical center. Patient pain scores were analyzed using a Friedman’s analysis of variance at a significance level of α = 0.05, and statistical significance between groups was elucidated using nonparametric post-hoc tests of significance between groups.

**Results:**

Pain scores at preprocedure, 2-month, 6-month, and 12-month follow-ups yielded significant differences. Post-hoc nonparametric analysis revealed pain scores at 2 months were significantly lower than preprocedure and at 6 months. Additionally, 47.5% of cases in this study went on to require a secondary procedure of the respective shoulder after their barbotage treatment.

**Conclusion:**

Upon analysis, utilization of barbotage as a treatment for calcific tendonitis of the shoulder appears to produce notable pain reduction in the short term (specifically at the 2-month follow-up), but begins to lose some efficacy over long-term evaluation. Additionally, a large portion of patients required further interventions of their shoulder, including corticosteroid injections, more barbotage, or surgery, raising further concerns over its long-term benefit.

Shoulder pain is one of the most common musculoskeletal issues found in the general population, with rotator cuff pathologies making up a large portion of these problems.^15^ Calcific tendinitis is a specific cause of rotator cuff inflammation and pain in which calcium (mainly in the form of hydroxyapatite) deposits in the subacromial space or within the rotator cuff, usually in the supraspinatus and infraspinatus tendons.^4^ This condition is estimated to impact 6.8% of patients with shoulder pain, with women affected at a slightly higher rate than men.^15^^,^^20^ When noninvasive treatments such as physical therapy and nonsteroidal anti-inflammatory drugs are ineffective at alleviating calcific tendinitis symptoms, “mini-invasive” procedures are often pursued, such as barbotage.

Barbotage consists of needle aspiration and subsequent lavage of calcium deposits in the shoulder, typically under the guidance of ultrasound.^2^^,^^17^ Use of barbotage for calcific tendinitis has been found to promptly reduce patient pain and increase shoulder function.^3^^,^^8^^,^^19^ Typically, the barbotage procedure itself is accompanied by a corticosteroid injection as well.^4^^,^^6^^,^^7^^,^^9^^,^^14^^,^^21^ Results have shown that barbotage with corticosteroid injection significantly improves Constant-Murley Score and decreased size of calcium deposit when compared to corticosteroid injection alone.^2^

Barbotage is a proven useful treatment method for calcific tendinitis, with multiple systematic reviews showing improvement in the majority of patients with low risk of complications.^11^^,^^13^ Despite the success of barbotage, there is still debate regarding the best treatment for calcific tendinitis.^16^^,^^17^ Other procedures, such as subacromial corticosteroid injections alone without barbotage, have shown similar efficacy at relieving calcific tendinitis symptoms in the long term, up to 5 years after initial treatment.^2^^,^^7^^,^^8^ Additionally, while barbotage often alleviates calcific tendinitis symptoms in the short term, some patients often experience symptom recurrence, with 1 systematic review citing a rate of 13% of patients (114 of 908) requiring further treatment.^11^ With these observations in mind, further analysis of patient outcomes following a barbotage procedure is indicated. This study aims to identify patient outcomes following barbotage treatment with corticosteroid injection for calcific tendinitis. We hypothesized that following barbotage treatment, patients would overall exhibit an improvement in their calcific tendinopathy pain at the follow-up points of 2, 6, and 12 months postprocedure.

## Methods

This was a retrospective study. A review of the records kept by Lahey Health and Medical Center (LHMC) was performed for data collection. We identified patients who underwent ultrasound-guided barbotage for the treatment of calcific tendinitis from January 2015 to December 2020 by interventional radiologists at our institution. This project was deemed Institutional Review Board Exempt Research status upon review by Lahey Clinic, Inc. Institutional Review Board (Study Number 20223162).

Eligibility criteria for this study required patients to be aged more than 18 years and who underwent barbotage for calcific tendinitis of the shoulder within the dates of January 2015 to December 2020 (January 2015 is the limit of current electronic medical records at LHMC). Per standard ultrasound-guided barbotage protocol, all patients underwent a corticosteroid injection as a component of their barbotage procedure. For all patients who fulfilled these criteria, preprocedural and postprocedural data were collected. Preprocedural data included demographics (age, sex, body mass index, and dominant hand), history of previous shoulder treatments (including previous corticosteroid injections), and prebarbotage pain scores of the affected shoulder (Table I). Postprocedure data involved evaluation of reported pain scores at the approximate time points of 2 months, 6 months, and 12 months after barbotage. Pain scoring was performed via interview pre-examination via a numeric scoring system (0-10, with 10 being the most severe pain possible). Additionally, any subsequent treatments to the shoulder, including corticosteroid injections, additional barbotage, or surgery, were also recorded.

**Table I:** Patient demographics and result totals.

Total unique patients	Total barbotage procedures	Total prebarbotage pain scores	Total 2-month follow-up pain scores	Total 6-month follow-up pain scores	Total 12-month follow-up pain scores
150	179	170	71	64	59

Sex	Mean BMI	Mean age	Dominant handedness	Side of barbotage	Previous ipsilateral shoulder treatment (including injection)
	Min BMI	Min age			
	Max BMI	Max age (y)			

58.7% female	28.7	55.3	78.8% right	55.8% right	43.0%
	19.1	22			
	49.4	96			

*BMI*, body mass index.

Pain scores were analyzed using a Friedman’s analysis of variance at a significance level of α = 0.05. Scores and demographic information were added to a secure Microsoft Excel spreadsheet. Statistical significance between groups was elucidated using nonparametric tests of significance between related samples. A Bonferroni correction of 0.05/6 = 0.008 was used as an alpha level for the 6 post-hoc tests performed. These analyses were completed using IBM SPSS statistics (IBM Corp., Armonk, NY, USA).

## Results

Between January 2015 and December 2020, 179 barbotage procedures in 150 patients were performed at LHMC. The average age of the patients at the time of the barbotage was 55.3 years (± standard deviation [SD] of 11.0 years). Of the cases, 58.7% (105/179) of the cases involved female patients. Overall patient mean body mass index was 28.7. Of these patients, 78.8% were right-handed. The majority of barbotage procedures were performed on the right shoulder (55.8%). A notable portion (43%) of patients had previously received ipsilateral shoulder treatment prior to their barbotage procedure, which included corticosteroids injections, previous barbotage, and surgery. Demographics information is displayed in Table I. Of the 179 procedures, 170 preprocedure notes, 71 2-month follow-up notes, 64 6-month follow-up notes, and 59 12-month follow-up notes were analyzed for pain score evaluation. While 95.0% of barbotage cases had a preprocedure pain score, pain scores were lost at an increasing amount at each subsequent follow-up time point (60.3% at 2 months, 64.3% at 6 months, and 67.0% at 12 months). This was due to patients missing follow-up appointments following barbotage.

**Figure 1:**
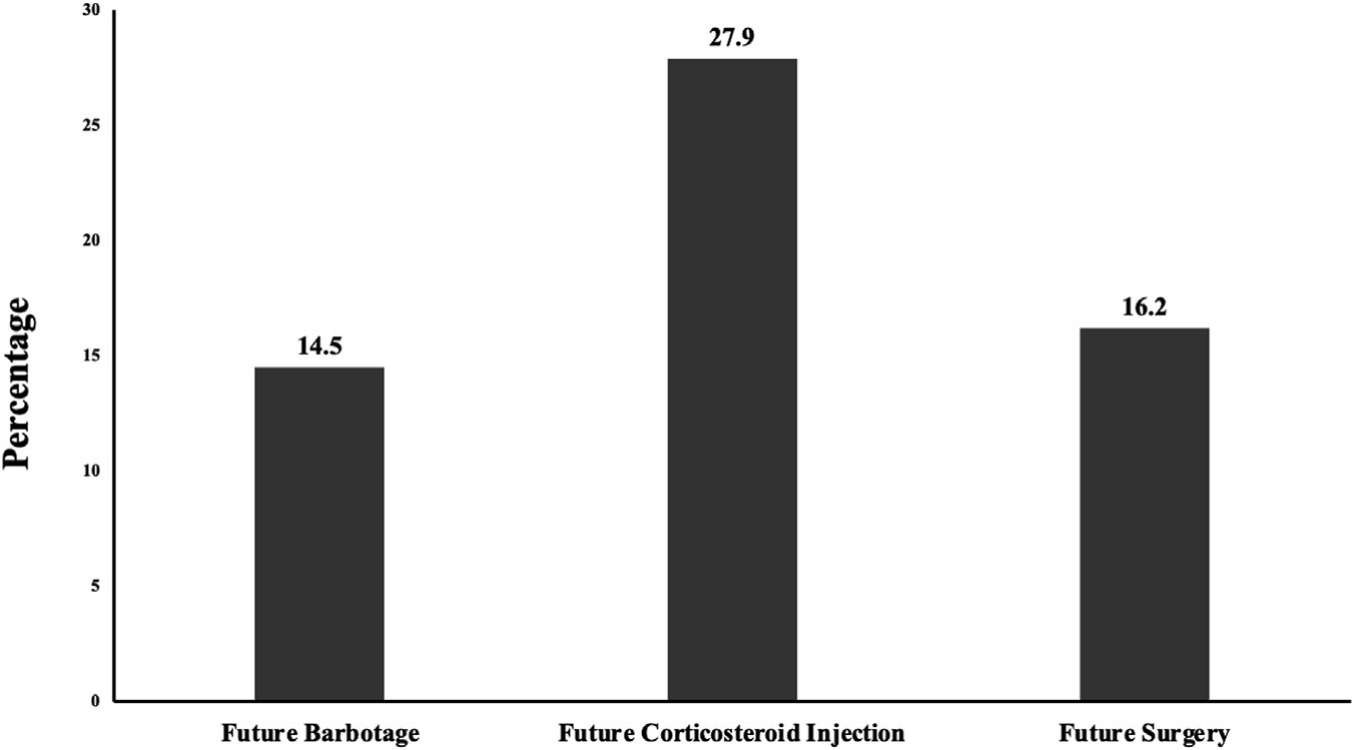
The percentage of barbotage patients who went on to undergo further treatment modalities for their calcific tendinitis. Total amount of cases that required further treatment to create these percentages included 26 cases involved subsequent barbotage treatment, 50 cases required corticosteroid, and 29 required surgery. The total number of cases that required any form of subsequent treatment modality was 85 of the 179 cases included in this study.

Additionally, following a barbotage procedure, 47.5% of patients went on to require a subsequent procedure for the respective shoulder. Procedures included corticosteroid injection, additional barbotage, or surgery. The average amount of time between barbotage and the next procedure was 300 days (± SD of 273 days). 14.5% of barbotage procedures required an additional barbotage at an average of 263 days (± SD of 164 days) after the initial procedure. 27.9% of barbotage procedures underwent a corticosteroid injection at an average of 355 days (± SD of 333 days) after the initial procedure. 16.2% underwent surgery following the initial barbotage at an average of 357 days (± SD of 194 days) after the initial procedure. These results are displayed in Figure 1.

**Figure 2:**
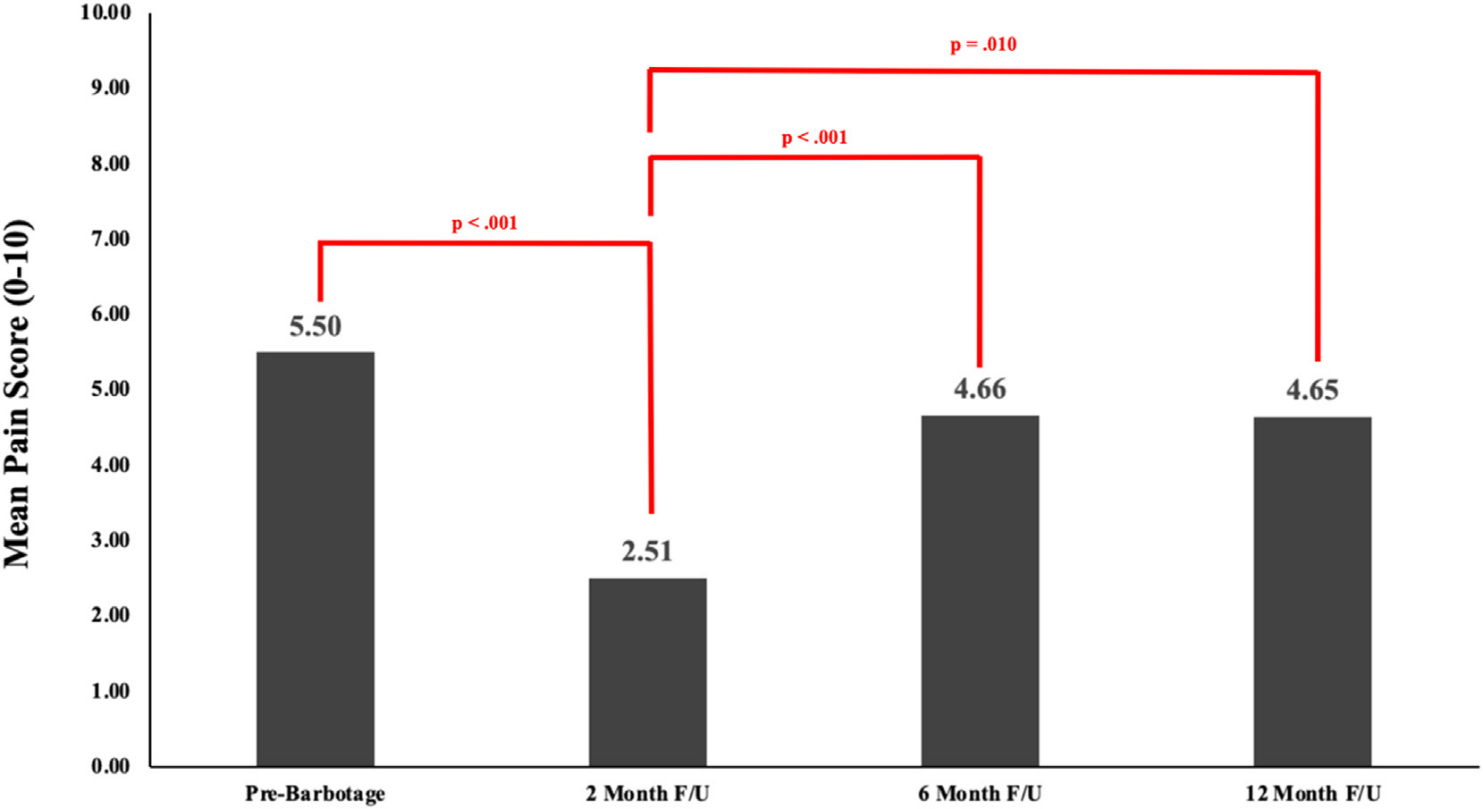
Mean pain scores at various intervals prior to and following barbotage. Of note using Friedman's analysis of variance with post-hoc analysis, pain scores at 2-month follow-up (0-10, with 10 being most severe pain) were found to be significantly lower than pain scores prebarbotage and at 6-month follow-up (follow-up shortened to F/U). A comparison between 2-month follow-up and 12-month follow-up was found to not be statistically significant when incorporating a Bonferroni correction. Overall, pain scores at evaluations further from the date of barbotage (at 6 months and 12 months) appear to be more similar to those at initial evaluation. The pain scores displayed omitted pain scores obtained following a secondary procedure.

**Figure 3:**
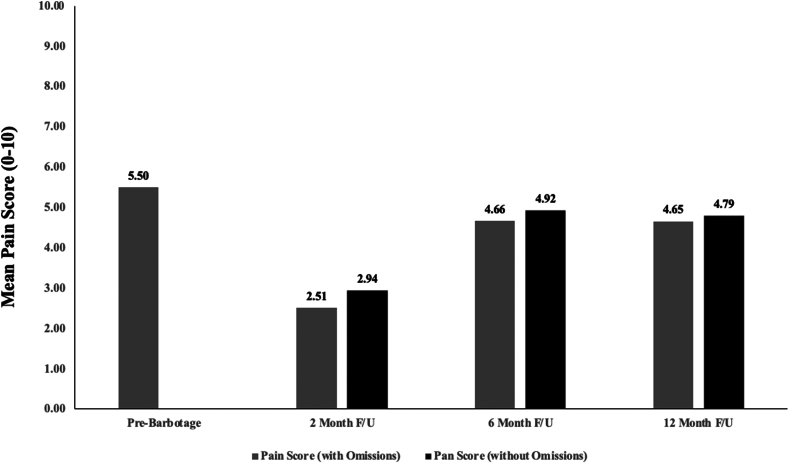
Mean pain scores with and without pain score omissions. Scores collected following a secondary procedure (including corticosteroid injection, subsequent barbotage, or surgery) of the ipsilateral shoulder were omitted in the omission groups (due to concern of confounding). These are presented in grey. Means without omissions are present in black. Paired student’s *t*-test showed no significant differences between the means of omitted vs. not omitted at each time point (2-month, 6-month, and 12-month follow-up). F/U is used as an abbreviation for “follow-up” in the display, and pain score refers to a 0-10 scale with 10 being the most severe pain.

A significant number of the secondary shoulder interventions (mentioned previously involving corticosteroid injection, subsequent barbotage procedure, or surgery) occurred within a year of the initial barbotage procedure. Due to this, pain score breakdown is reported in 2 different representations. Mean pain score (0 to 10, 10 being the most severe) prebarbotage was 5.50 (± SD of 2.71). When all collected pain scores were used, 2-month, 6-month, and 12-month follow-ups were 2.94 (± SD of 2.96), 4.92 (± SD of 2.70), and 4.79 (± SD of 2.84), respectively. When pain scores collected after (including the day of) a secondary shoulder intervention were omitted, 2-month, 6-month, and 12-month follow-ups scores were 2.51 (± SD of 2.79), 4.66 (± SD of 2.71), and 4.65 (± SD of 3.10), respectively. These results are displayed in Figure 2. Comparison between the pain score data with omissions vs. without can be found in Figure 3. These ommisions were deemed necessary for statistical analysis to minimize confounding factors to pain scores.

A Friedman’s analysis of variance test of data excluding pain scores obtained following a secondary procedure found a *P* value of <.001. Post-hoc nonparametric analyses between related samples found the 2-month follow-up pain score to be significantly smaller than the pain scores of preprocedure and 6-month follow-up time points (<0.001 and <0.001). A difference was also noted between the time points of 2-month follow-up and 12-month follow-up (*P* = .010), however was not significant when taking into account Bonferroni correction.

## Discussion

Upon analysis of LHMC patients, utilization of barbotage as a treatment option for calcific tendonitis of the shoulder appears to produce notable pain reduction in the short term (specifically at the 2-month mark following the procedure) but begins to lose efficacy over more long-term evaluations, particularly by 6 months. Additionally, a substantial proportion of barbotage treatments eventually require further procedures, whether this be corticosteroid injections, additional barbotage, or surgical intervention.

Similar to the results of this study, a symptom recurrence following barbotage for calcific tendinitis was found in a randomized control trial.^7^ This manifested around 3 months, but these symptoms were found to be temporary, and typically resolved in subsequent follow-ups at 6 months, 12 months, and 5 years. Comparable results were also found in another study, with symptoms recurring transiently between postprocedure weeks 5-28 weeks.^9^

The transient nature of the return of symptoms appears potentially similar to the results found in this analysis. Overall, the barbotage cases of both of these studies had significant improvement in their calcific tendinitis symptoms by 12 months postbarbotage (reported via Constant Score, Shoulder Pain, and Disability Index, respectively).^7^^,^^9^ Similar to these 2 studies, others have also reported continued improved shoulder functionality at 12 months postbarbotage, however without mention of a transient symptom recurrence.^14^^,^^19^ While our results also depict a significant increase in pain scores at 6 months but not at 12 months, it is important to appreciate there was still a notable, yet not statistically significant, increase in pain scores at 12 months compared to 2-month follow-up as well.

Overall, it appears ubiquitous that barbotage provides a degree of symptom relief for calcific tendinitis in most patients.^3^^,^^6^^,^^7^^,^^9^^,^^11^^,^^14^^,^^19^ Despite this, there is variability in the degree of success. In a systematic review of barbotage for calcific tendinitis, it was determined that there is insufficient evidence to conclude that ultrasound-guided needle lavage is a superior treatment option to other methods of addressing calcific tendinitis.^23^ This insufficiency was attributed to variations in treatment approaches and lack of high-quality evidence. The observed reductions in pain, disability, and calcification size and improvement in degree of movement found in the studies reviewed could not be conclusively attributed to ultrasound-guide needle lavage alone.

Corticosteroid injections are another proven effective treatment option for calcific tendinitis.^5^^,^^18^ In addition to being a helpful treatment option, corticosteroids also provide a lower cost benefit compared to barbotage, as bony landmarks can be used rather than imaging modalities for injection guidance.^10^ While one study has already performed a randomized control trial to compare corticosteroids vs. barbotage directly, this study contained only 48 subjects, and found that both treatment modalities improved calcific tendinitis symptoms by 12 months postbarbotage.^8^ With these results in mind, further direct evaluation is warranted. Future study objectives for LHMC include a larger randomized control trial to better assess whether barbotage with corticosteroid injection offers superior efficacy to corticosteroid injections alone.

Additionally, the need for secondary treatment following barbotage seems to have varied between studies. In a systematic review to assess the efficacy of barbotage for calcific tendinitis, it was found that across 13 studies there was a 13% retreatment rate (114 of 908 patients) following initial barbotage. In most cases, the second treatment was an additional barbotage, with only around 1% (9 of 908 patients) requiring surgery.^11^ This notably differs from the rates seen in our cases, with 47.5% requiring further treatment; 27.9% required subsequent barbotage and 16.2% required surgery.

Comparable rates of postbarbotage interventions were found in a prospective study of 123 patients who underwent barbotage and followed for 6 months after.^6^ Within this study, 42% of patients had persistent symptoms that required 2 or more treatments with lavage and corticosteroid infiltration of the bursa. Postbarbotage surgery rates were not mentioned in this study. A potential explanation for the differences in retreatment is the phase at which barbotage is performed. It has been suggested in previous literature that treating patients during the formative and resting phases of calcific tendinitis rather than the resorptive phase can be ineffective at improving symptoms.^11^^,^^22^ Evidence is limited, however, and further comparison of treating between these phases is necessary. Additionally, evidence suggests that somewhere between 30% and 80% of rotator cuff calcific tendinitis is successfully managed with conservative treatment alone.^12^ Furthermore, even when calcific tendinitis symptoms extend past 8 weeks and are deemed chronic, symptoms may still spontaneously heal on their own.^1^ With this in mind, it is worth considering that other studies with higher success rates of barbotage may overestimate its efficacy, specifically that some cases deemed a success from barbotage may have resolved on their own with continued conservative measures.

Additionally, the consistency of patient follow-up likely played an impact in results. This study involved 179 unique barbotage procedures in total. While the vast majority underwent initial evaluation prior to barbotage treatment (95%), this proportion began to dwindle with each subsequent follow-up. Of the 179 cases, 40% (71 cases) had a 2-month follow-up, 36% (64 cases) had a 6-month follow-up, and 33% (59 cases) had a 12-month follow-up. With more than 50% of pain scores missing from any given follow-up time point, it is reasonable to assume that trends in pain scores could vary with a larger proportion of pain scores reported. This proportion of missing scores contrasts other studies, which had far more successful follow-up rates.^6^^,^^8^^,^^9^ While it is impossible to determine with complete certainty, it is plausible patients who had more significant improvement from their initial barbotage treatment could be more likely to decline follow-up appointments, making mean pain scores disproportionately lower than in actuality.

### Limitations

In addition to being a retrospective chart review and the associated biases that come with this method of research, there are several notable limitations to this study. First, as previously mentioned, a large proportion of patients were lost to follow-up, potentially skewing pain score results. Additionally, due to the impacts of COVID-19 on clinical practice, overall evaluation of patients at follow-up appointments was noticeably impacted, including consistent evaluation of upper extremity functionality values following barbotage. This includes useful scores such as Disabilities of the Arm, Shoulder, and Hand questionnaire, as well as other measurements, such as shoulder flexion/extension, abduction/adduction, and internal/external rotation. Unfortunately, these values were not reported frequently enough in barbotage cases to be used in this study. All of these measurements and other similar variations (such as American Shoulder and Elbow Surgeons Shoulder Score, Western Ontario Rotator Cuff Index, Shoulder Pain, and Disability Index) would have been useful in further examining overall improvements in shoulder functionality following treatment, and thus were frequently used in other studies involving barbotage.^8^^,^^9^^,^^14^

## Conclusion

Our hypothesis was that barbotage would alleviate shoulder pain at the time points of 2 months, 6 months, and 12 months postprocedure. Results of this study indicate that while barbotage for calcific tendinitis of the rotator cuff can provide short-term pain relief, its long-term benefits (specifically at 6 and 12 months postbarbotage) appear limited. Additionally, a large portion of barbotage cases required further treatment, with subsequent barbotage, corticosteroid injection, or surgery. These results appear to contrast many other studies published examining barbotage, which show symptom improvement up until at least 1 year. To further investigate this variation, Lahey Hospital and Medical Center plans to conduct a large, randomized control trial directly comparing barbotage to corticosteroid injection, to better understand how these 2 preferred treatment modalities for calcific tendinitis of the rotator cuff impact symptoms.

## Disclaimers:

Funding: No funding was disclosed by the authors.

Conflicts of interest: The authors, their immediate families, and any research foundation with which they are affiliated have not received any financial payments or other benefits from any commercial entity related to the subject of this article.
